# Antiphospholipid autoantibodies in Lyme disease arise after scavenging of host phospholipids by *Borrelia burgdorferi*

**DOI:** 10.1172/JCI152506

**Published:** 2022-03-15

**Authors:** Peter J. Gwynne, Luke H. Clendenen, Siu-Ping Turk, Adriana R. Marques, Linden T. Hu

**Affiliations:** 1 Department of Molecular Biology and Microbiology, Tufts University School of Medicine, Boston, Massachusetts, USA.; 2 Laboratory of Clinical Microbiology and Immunology, National Institute of Allergy and Infectious Diseases (NIAID), NIH, Bethesda, Maryland, USA.

**Keywords:** Infectious disease, Microbiology, Diagnostics, Immunoglobulins, Molecular biology

## Abstract

A close association with its vertebrate and tick hosts allows *Borrelia burgdorferi*, the bacterium responsible for Lyme disease, to eliminate many metabolic pathways and instead scavenge key nutrients from the host. A lipid-defined culture medium was developed to demonstrate that exogenous lipids are an essential nutrient of *B*. *burgdorferi*, which can accumulate intact phospholipids from its environment to support growth. Antibody responses to host phospholipids were studied in mice and humans using an antiphospholipid ELISA. Several of these environmentally acquired phospholipids including phosphatidylserine and phosphatidic acid, as well as borrelial phosphatidylcholine, are the targets of antibodies that arose early in infection in the mouse model. Patients with acute infections demonstrated antibody responses to the same lipids. The elevation of antiphospholipid antibodies predicted early infection with better sensitivity than did the standardized 2-tier tests currently used in diagnosis. Sera obtained from patients with Lyme disease before and after antibiotic therapy showed declining antiphospholipid titers after treatment. Further study will be required to determine whether these antibodies have utility in early diagnosis of Lyme disease, tracking of the response to therapy, and diagnosis of reinfection, areas in which current standardized tests are inadequate.

## Introduction

With an average genome of approximately 1.3 mbp (1), many aspects of cellular metabolism are minimized or absent in *Borrelia burgdorferi*. Such an evolutionary genome reduction is possible because *B*. *burgdorferi* lives in close association with vertebrate and tick hosts (2), which are parasitized for many common metabolites synthesized de novo by other bacteria. Transport and scavenging mechanisms therefore replace many common synthetic pathways (3–5).

Characterized pathways of lipid metabolism are limited to the synthesis of phosphatidylcholine (PC), phosphatidylglycerol (PG), and 2 monogalactosyl lipids: galactosylcholesterol and galactosyldiacylglycerol (6). The acyl lipids derive from a common pathway in which glycerol-3-phosphate is twice acylated to form phosphatidic acid (PA), which is modified with choline, glycerol, or galactose (7, 8). Precursors for all membrane constituents are presumed to be scavenged from the host as *B*. *burgdorferi* lacks apparent synthetic pathways for fatty acids, cholesterol, and choline. Although the membrane of in vitro–cultured *B*. *burgdorferi* appears to be mostly composed of these 4 lipid species, chromatographic fractionation suggests a complex mixture of at least 11 components, most of which are unidentified (9).

The dependence on environmentally acquired fatty acids was determined with radiolabeling in *B*. *burgdorferi* (10) and the related *Borrelia hermsii* (11). In the former, the fatty acid composition of the membrane approximates that of the medium (12). Given this observation, and that the otherwise well-conserved *fad* system of fatty acid translocation is also absent from *Borrelia* (1), uptake into the periplasm is likely to be passive via diffusion across the membranes. This phenomenon is likely facilitated in spirochetes by their lack of an exopolysaccharide coat, which leaves them unusually accessible to hydrophobic moieties. Similarly, cholesterol acquisition has been shown to be mediated by the fusion of host-derived membrane vesicles with *B*. *burgdorferi* (13).

Although the majority of characterized antibodies against *B*. *burgdorferi* recognize protein antigens, anti-lipid antibodies also develop during infection. Antibodies against both galactosylcholesterol and galactosyldiacylglycerol are raised in Lyme disease and remain elevated after treatment (14). These antibodies have been shown to cross-react with mammalian gangliosides (15), and this reaction has been suggested as a possible cause of neurologic symptoms of borreliosis. Lipids also stimulate the innate immune response, with natural killer T (NKT) cells being activated by the galactosyldiacylglycerol (16) of *B*. *burgdorferi* to produce immunostimulatory IFN-γ, which is responsible for the recruitment of macrophages and, ultimately, the clearing of infection (17, 18).

Although not widely studied, anti-lipid antibodies are described in other infectious diseases (19–21) as well as in systemic autoimmune conditions (22, 23). Syphilis, caused by the related spirochete *Treponema pallidum*, is diagnosed by serology to detect antibodies against either treponemal (lipoprotein) or nontreponemal (lipid) epitopes. The nontreponemal rapid plasma regain (RPR) or venereal disease research laboratory (VDRL) tests use an antigen mixture of cardiolipin (CL), lecithin, and cholesterol, of which CL is thought to be the most seroreactive (24). CL is found in both the mitochondrial membranes of the host and the membranes of infecting treponemes (25). The high titers seen during an active infection are thought to result from CL and other lipids liberated from damaged host cells as well as those presented directly by the bacteria (26). Quantitative measurement of the nontreponemal antibodies, which decline within 6 to 12 months of bacterial clearance, allows for the monitoring of treatment success (27). This quantitation informs clinical decisions regarding the need for further treatment. In the absence of an equivalent test for Lyme disease, approaches to the management (and even the definition) of persistent symptoms vary widely (28).

Serology is the mainstay in the clinical diagnosis of Lyme disease (29). CDC-recommended standard testing algorithms use a 2-stage protocol of an ELISA followed by either Western blotting or a second ELISA (30). Current tests are accurate in the detection of established infections, but during the earliest stages of infection, most antibody tests are only 50%–60% sensitive (31). Additionally, these antibody tests can remain positive for decades after antibiotic treatment (32), limiting their usefulness in determining treatment success or identifying reinfection. To identify antiphospholipid antibodies with potential for use as diagnostic markers of Lyme disease, the incorporation of diverse phospholipids by *B*. *burgdorferi* was investigated. This was followed with a survey of antiphospholipid antibodies in *B*. *burgdorferi*–infected mice and the identification of an antibody panel able to discriminate between healthy controls and patients with Lyme disease. Unlike current tests, the antibodies identified also allowed for monitoring of the response to therapy, with titers declining in the year following treatment.

## Results

### B. burgdorferi is a lipid auxotroph.

**Figure 1:**
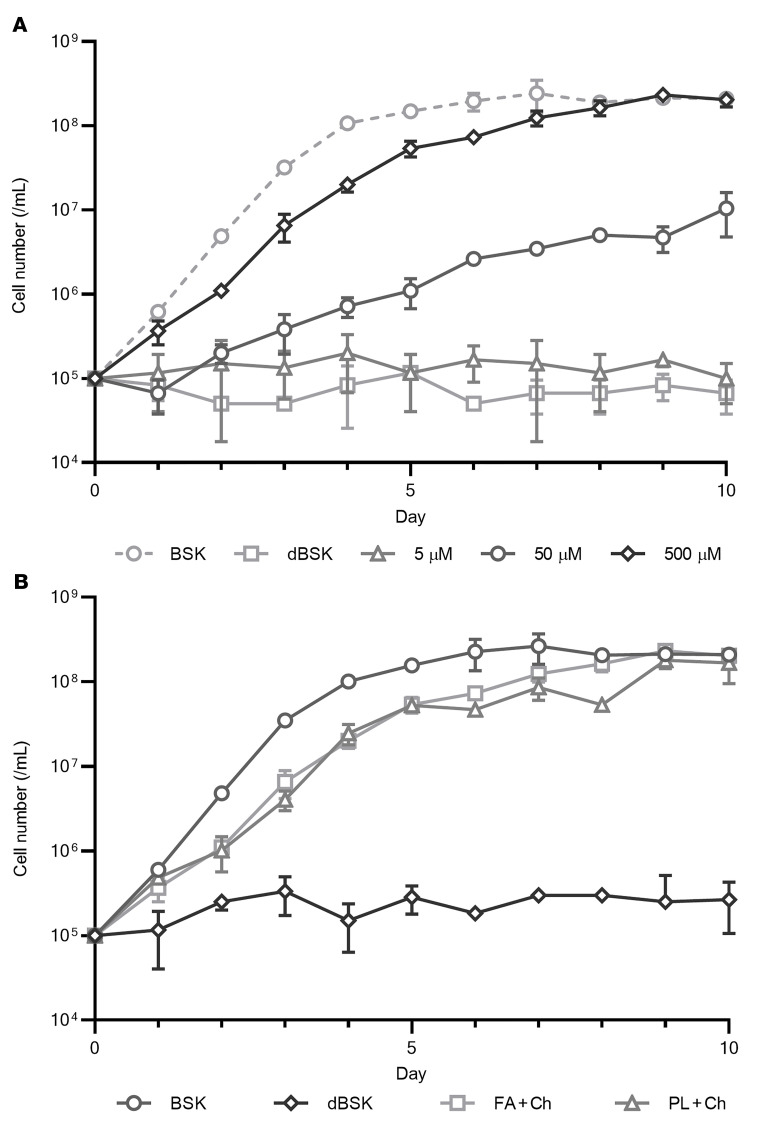
Growth of *B*. *burgdorferi* is dependent upon exogenous lipids. (**A**) Growth of *B*. *burgdorferi* in lipid-free medium supplemented with fatty acids and cholesterol (2:2:1 palmitic acid/oleic acid/cholesterol) to a final concentration of 5–500 μM. At 500 μM, cell density equivalent to that of the unmodified BSK medium was reached. No growth was observed in the absence of lipid (dBSK only). (**B**) Equivalent growth over 10 days of *B*. *burgdorferi* in medium supplemented with fatty acids (FA) (200 μM each of palmitic and oleic acids) and phospholipids (PL) (100 μM each of PG and PC). Cholesterol (Ch) was present at 100 μM in all media. Data plotted in **A** and **B** show the mean of 3 biological replicates, with error bars indicating the SD.

*B. burgdorferi* is unable to grow in a lipid-depleted growth medium (delipidated BSK [dBSK]), with growth being restored by supplementation with fatty acids and cholesterol (Figure 1A). A standard Barbour-Stoenner-Kelly II (BSK) growth medium was stripped of lipids by organic extraction, yielding a medium unable to support the growth of *B*. *burgdorferi*. On the basis of previous analyses of borrelial membranes (6, 9), cholesterol and fatty acids were supplied at a molar ratio of 1:4, with the fatty acids consisting of an equal mix of palmitic (16:0) and oleic (18:1) acid. Upon supplementation with lipids, the growth was restored in a concentration-dependent manner. Darkfield microscopy confirmed that the cells cultured in lipid-supplemented dBSK had a morphology and motility similar to those of cells cultured in standard BSK.

In addition to fatty acids, intact phospholipids can be used as a lipid source for growth (Figure 1B). A similar growth curve was seen with fatty acids (200 μM palmitic acid, 200 μM oleic acid) and an equivalent concentration of phospholipids (100 μM PG, 100 μM PC). Cholesterol was included at 100 μM in all cultures.

### B. burgdorferi accumulates environmental phospholipids.

**Figure 2:**
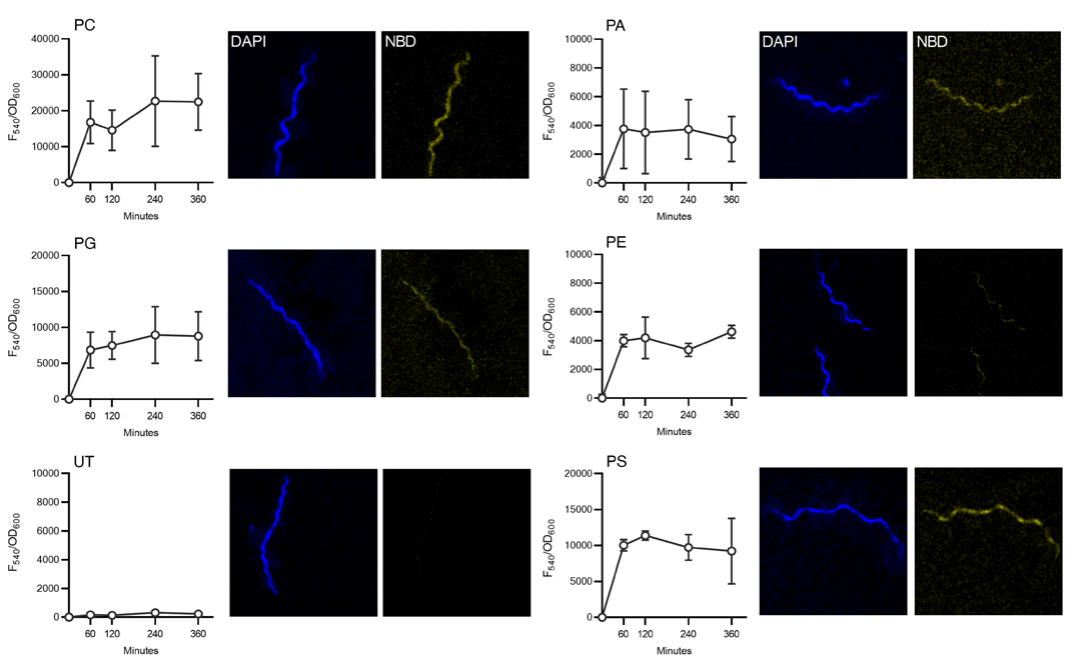
Uptake of fluorescence-labeled phospholipids by *B*. *burgdorferi*. The canonical phospholipids of *B*. *burgdorferi* PC and PG, as well as the noncanonical membrane components PA, PE, and PS, were acquired from the medium. Untreated cells (UT) did not fluoresce in the NBD channel. Cells were incubated in dBSK plus 25 μM NBD-labeled phospholipid analogs over a 6-hour period, and then imaged by fluorescence microscopy at ×63 magnification. DAPI stained the nucleic acids nonspecifically. DAPI (Ex = 405 nm, detector = 410–466 nm) and NBD (Ex = 470 nm, detector 550–600 nm) channels were acquired sequentially. Fluorescence intensity (Ex = 465 nm, Em = 540 nm, normalized to OD_600_) was quantified by fluorimetry. Graphs plot the mean of 3 biological replicates, with error bars indicating the SD. Images are representative of biological triplicate experiments.

**Figure 3:**
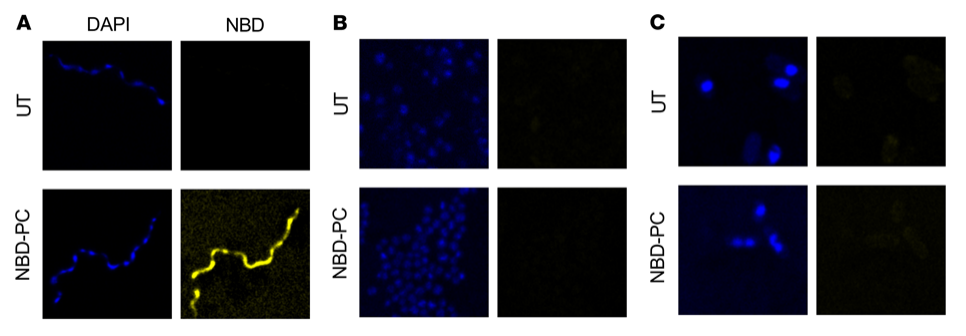
The membrane of *B*. *burgdorferi* is more accessible than that of other species. *B*. *burgdorferi* (**A**), *S*. *aureus* (**B**), and *E*. *coli* (**C**) were all incubated with 25 μM NBD-PC for 4 hours. Only *B*. *burgdorferi* acquired the fluorescent phospholipid. DAPI stained nucleic acids nonspecifically. DAPI (Ex = 405 nm, detector = 410–466 nm) and NBD (Ex = 470 nm, detector 550–600 nm) channels were acquired sequentially at a magnification of ×63. Images are representative of biological triplicate experiments.

Given the utilization of phospholipids from the growth medium, their uptake was assayed using fluorescent (nitrobenzoxadiazole-labeled [NBD-labeled]) phospholipid analogs. Although *B*. *burgdorferi* can synthesize PC and PG from precursors (7), these previously identified *B*. *burgdorferi* phospholipids can also be acquired intact from the growth medium (Figure 2). Further, 3 phospholipids that, to our knowledge, have not previously been identified in *B*. *burgdorferi* (phosphatidylethanolamine [PE], phosphatidylserine [PS], and PA) were also incorporated into borrelial membranes when present in the medium (Figure 2). Under the same conditions, NBD-PC did not enter the membranes of either *E*. *coli* or *S*. *aureus* (Figure 3), suggesting that this phenomenon may be particular to *Borrelia*.

Incorporation of all phospholipids occurred within 1 hour and was largely constant over 6 hours, indicative of a rapidly established equilibrium between cell membranes and the medium. The disparity in intensity between the 5 fluorophores may indicate that the borrelial membrane was differently accessible to the various lipid species, possibly as a function of the lipoprotein coat. However, the solution dynamics of the assayed phospholipids is also likely to affect their accessibility. Their solubility and critical micelle concentrations in the dBSK medium were not determined but are likely to be variable across the group.

**Figure 4:**
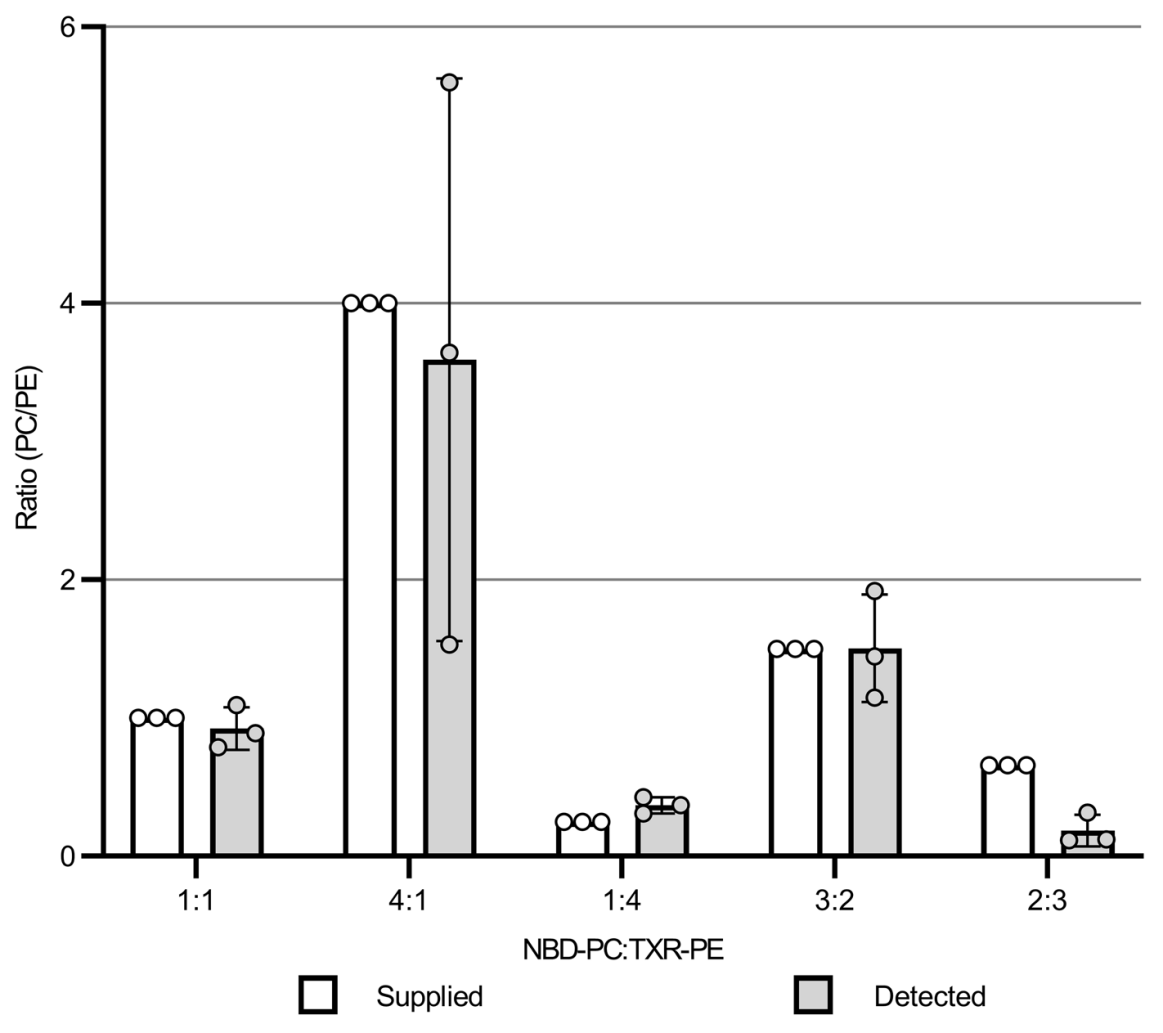
The borrelial membrane reflects the surrounding medium. *B*. *burgdorferi* was incubated with 2 phospholipid fluorophores — NBD-PC and Texas Red–PE (TXR-PE) — at different ratios for 4 hours. The ratio of fluorescence intensity in washed cells (F_540_/F_625_) closely matched the ratio of fluorophores available in the culture medium (μM NBD-PC vs. μM TXR-PE). The total concentration of the phospholipid was 25 μM for all conditions. Bars represent the mean of 3 biological replicates, with error bars indicating the SD.

**Figure 5:**
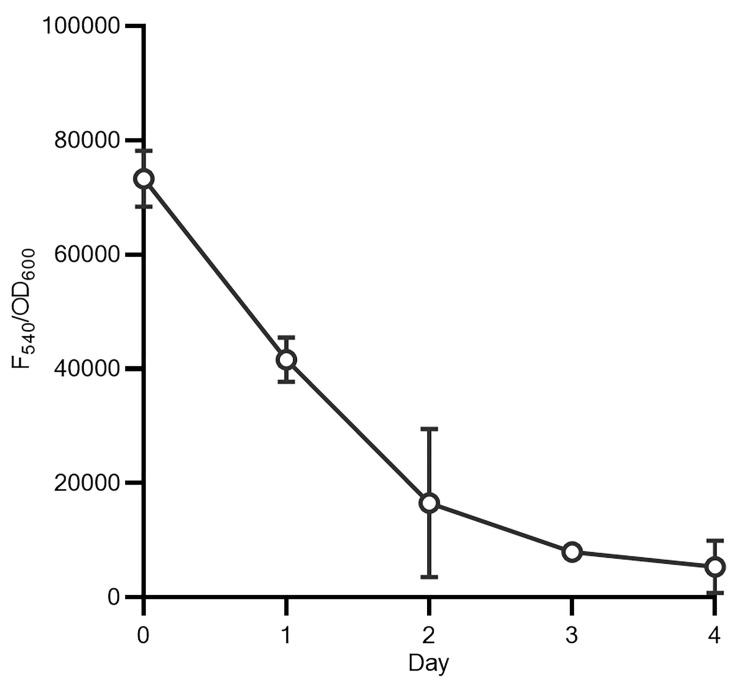
Phospholipids are retained in the membrane in the absence of an environmental source. *B*. *burgdorferi* was labeled with NBD-PC in dBSK medium for 4 hours, washed, and resuspended in rich medium. Fluorescence intensity (F_540_/OD_600_) was measured daily and declined with a half-life of approximately 24 hours, with 56% of the day 0 fluorescence intensity on day 1 and approximately 7% on day 4. Data points represent the mean of 3 biological replicates, with error bars indicating the SD.

Using a competition assay between phospholipids with 2 different fluorophores (NBD-PC and Texas Red–PE), we show that phospholipids entered the membrane at approximately the same relative concentration as in the medium (Figure 4). This is indicative of nonspecific uptake and is consistent with a model of random diffusion into the membrane. Fluorescence was detectable for up to 4 days after the initial labeling, with a half-life of approximately 1 day (Figure 5). Although the decline in signal could be a result of either degradation or membrane turnover (as fluorophores are shed to the medium), the *B*. *burgdorferi* genome predicts neither phospholipases nor fatty acid catabolic pathways, suggesting that degradation was unlikely.

### Antiphospholipid antibodies are raised during murine infection.

The seroreactivity of these noncanonical membrane components was assessed by ELISA using a mouse model of *B*. *burgdorferi* infection. Infected (4 weeks) and naive mouse sera were probed for antibodies against the phospholipids PG, PC, PA, PS, PE, and the PG dimer CL. Galactosylcholesterol antibodies, which have been extensively studied in Lyme disease (14, 33), were also assayed as a reference. Mice were infected for 28 days before the collection of serum, at which point they typically manifest active joint disease as well as IgG and IgM antibody responses (34).

**Figure 6:**
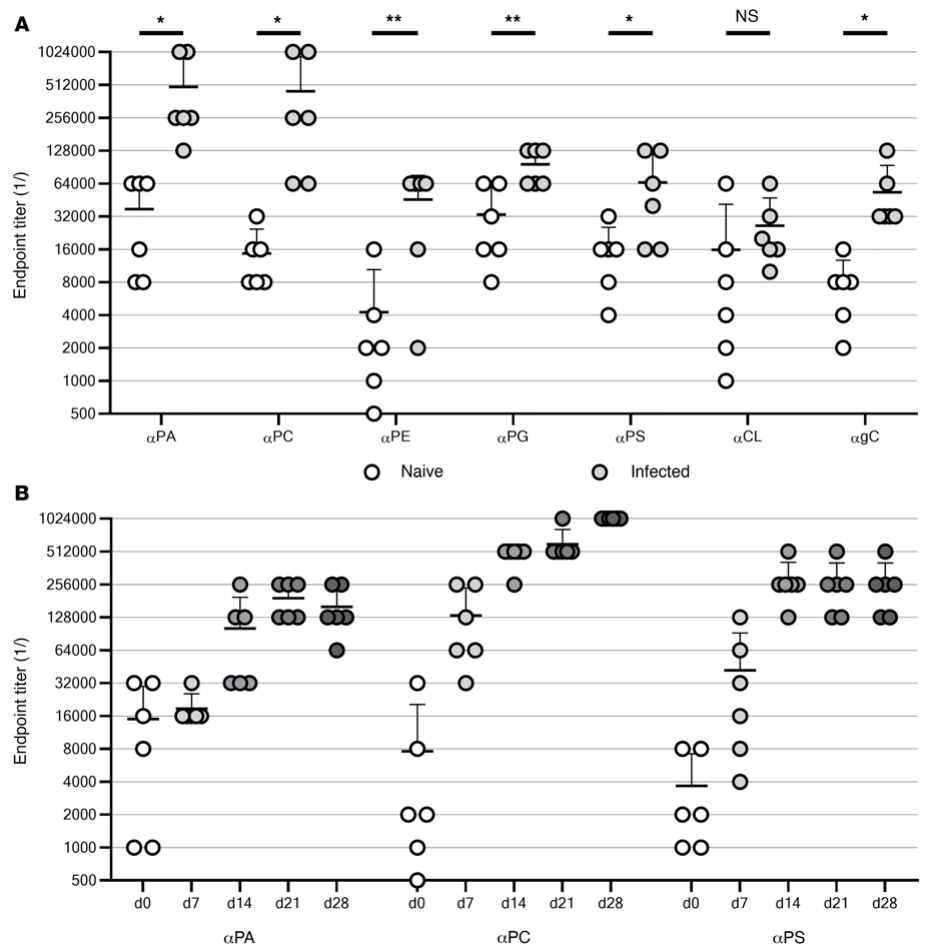
Anti-lipid antibodies are raised in a mouse model of *B*. *burgdorferi* infection. (**A**) Endpoint titers were determined for 7 antibodies (anti-PA [αPA], αPC, αPE, αPG, αPS, αCL, and anti-galactosylcholesterol [αgC]) in naive and infected 4-week-old C3H mice. Data (black lines indicate the mean of 6 replicates, and error bars indicate the SD) are plotted on a log_2_ scale to reflect binary dilution series. *P < 0.05 and **P < 0.01, by unpaired, 2-tailed *t* test. (**B**) In a different group of 6 mice, 3 antibodies were measured over the course of a 28-day (d) infection. Data are plotted as in **A**.

After 4 weeks of infection, antibodies were raised against 5 of the 6 assayed phospholipids, as well as galactosylcholesterol (Figure 6A). The antibody responses varied in magnitude, with post-infection titers of anti-PC and anti-PA being the greatest. Anti-PC and anti-PA also showed the highest relative increases from the naive levels at approximately 30- and approximately 13-fold, respectively. Although the final titer of anti-PE was modest (reaching about the same level as anti-PA and anti-PG in the naive mice), this represented a 10-fold increase from the lowest basal titer of the lipids assayed. Increases in anti-PS (~4-fold) and anti-PG (~3-fold) were smaller, but for every antibody except anti-CL, the comparison of naive and infected samples was statistically significant (*P <* 0.05).

To investigate the kinetics of the antiphospholipid responses, 6 additional mice were serially sampled over the first 4 weeks of infection (Figure 6B). Three antibodies (anti-PA, anti-PC, and anti-PS) were measured at each of 5 time points, with all 3 showing a similar pattern of induction. Titers begin to increase by day 7 and increased most dramatically between days 7 and 14. For 2 of the antibodies, the endpoint titers were stable over the later time points, suggesting that a peak was reached within 14 days (for anti-PS) or 21 days (anti-PA). Anti-PC continued to rise out to day 28 and may have been found to reach higher titers with prolonged infection.

### Antiphospholipid antibodies in human serum.

Human serum samples were probed for IgG antibodies against 4 of the phospholipids assayed above: PA, PC, PS, and CL. In order to mimic a higher-throughput diagnostic assay of the kind used in clinical settings, these immunoassays were performed at a single dilution, with the OD normalized to a panel of 12 uninfected controls run in parallel. Naive, untreated, and post-treatment groups each comprised 12 individuals (Supplemental Tables 1, 2, and 4). An additional group consisting of sera from 12 patients with syphilis (RPR- and treponemal IgG–positive) was used to assess the cross-reaction of the antiphospholipid tests. Under the assay conditions tested, no significant difference was found between the syphilis and control sera (Supplemental Figure 1; supplemental material available online with this article; https://doi.org/10.1172/JCI152506DS1).

**Figure 7:**
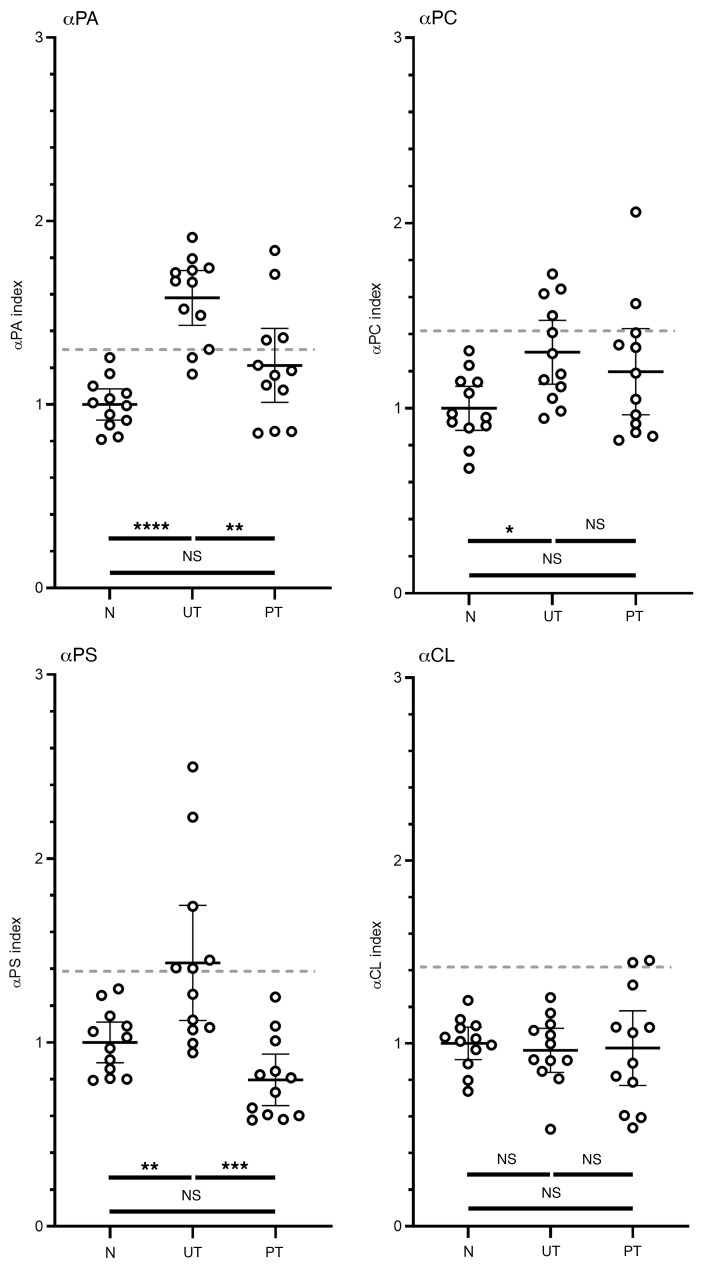
Antiphospholipid antibodies in human sera during infection. IgG anti-PA, anti-PC, and anti-PS were raised during infection, while anti-CL was not. Untreated and post-treatment (PT) groups were compared with the naive controls (N). Each group contained 12 individuals, with black lines representing the mean of the group and error bars the SD. All samples were normalized to the average of at least 6 naive controls run in parallel. An index of greater than 1 indicates that the antibody titer was above the level of that in the naive controls. Gray dashed lines represent the cutoff value (the mean of the naive group + 2.291 SDs) above which a sample was considered positive. *P < 0.05, **P < 0.01, ***P < 0.001, and ****P < 0.0001), by Tukey’s test for multiple comparisons.

Sera from 12 untreated individuals were collected at the time of diagnosis, 1 to 75 days after the onset of symptoms (mean = 17.8 days, median = 9.0 days). This time span includes the early stages of infection, when existing diagnostic antibodies have low sensitivity (31): 5 samples were collected 0–7 days after diagnosis, 4 samples 8–14 days after diagnosis, and 3 samples more than 21 days after diagnosis. The titers of 3 of the 4 antibodies assayed were significantly different (*P <* 0.05) in the control and untreated sera (Figure 7). As in the mouse model, anti-CL titers were unchanged during human infection. Treated sera were collected from 12 individuals at least 1 year (1–13 years, mean = 6 years, median = 5 years) from the initial diagnosis and treatment of Lyme disease, with all samples remaining positive by standardized two-tier (STT) testing.

The cutoff value, which defined a positive sample, was established using a panel of 12 negative control samples from healthy donors. The 3 phospholipids that were elevated during infection were assessed for diagnostic value using a cutoff value equal to the mean of the naive controls plus 2.291 SDs. This was derived using the method described by Frey et al. (35). In the untreated group, antiphospholipid titers predicted infection in 10 of 12, 4 of 12, and 6 of 12 samples for anti-PA, anti-PC, and anti-PS, respectively. If the 3 tests are combined as a panel and elevation of any single antibody constitutes a positive result, the diagnostic efficiency increased to 11 of 12. By the same definition, only 4 of the post-treatment samples were positive: 2 were elevated for anti-PC and anti-PA and 2 for anti-PA alone. Two of the patients with syphilis (16.7%) passed the cutoff for anti-PA only; it may be notable that these 2 patients also had the lowest treponemal IgG titers (Supplemental Table 4).

**Figure 8:**
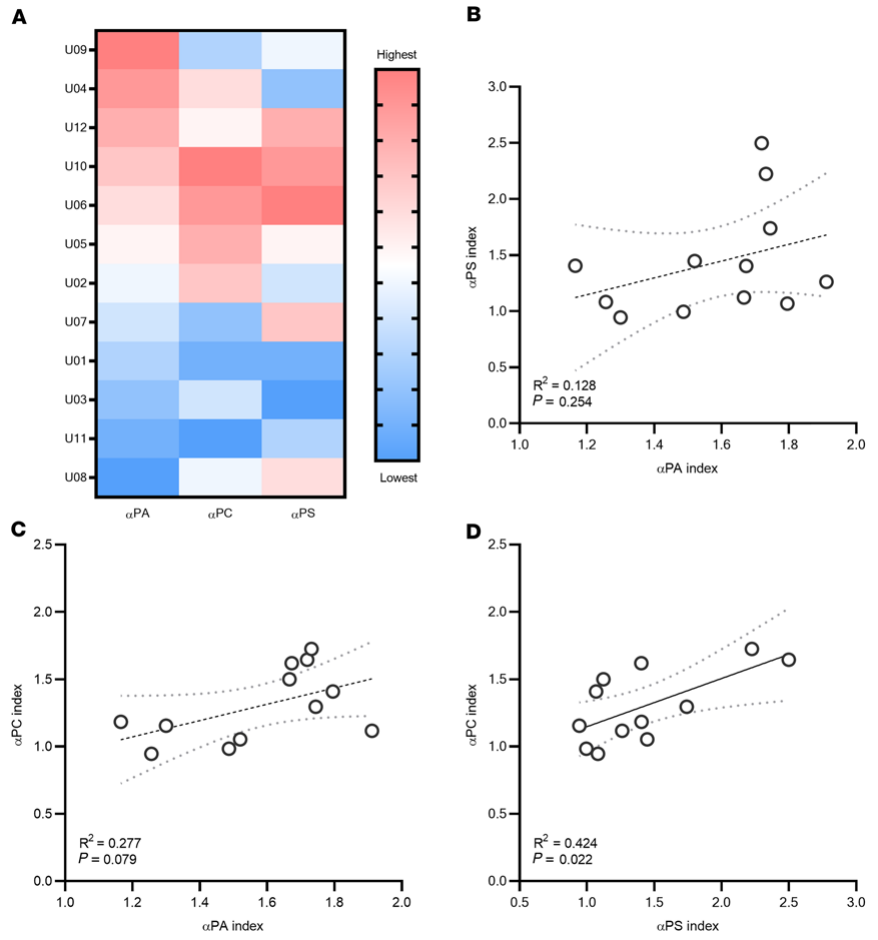
Independence of the 3 antibody responses. (**A**) Heatmap showing the rank score for each elevated antibody in each serum sample. (**B**–**D**) Linear regression analyses for pairwise comparisons of the 3 antibody responses. Only anti-PC versus anti-PS was significant, as determined by unpaired, 2-tailed *t* test (*P <* 0.05). Trend lines are shown in black and 95% CIs in gray dashed lines.

To assess whether the 3 antibodies arise independently, the pairwise correlations were analyzed by linear regression (Figure 8) and found that only anti-PC and anti-PS were significantly correlated. Further, each individual was ranked from 1 to 12 by their responses to each of the phospholipids. While some patients were consistently high or low across the 3 antibodies assayed, others showed variable responses to each.

**Figure 9:**
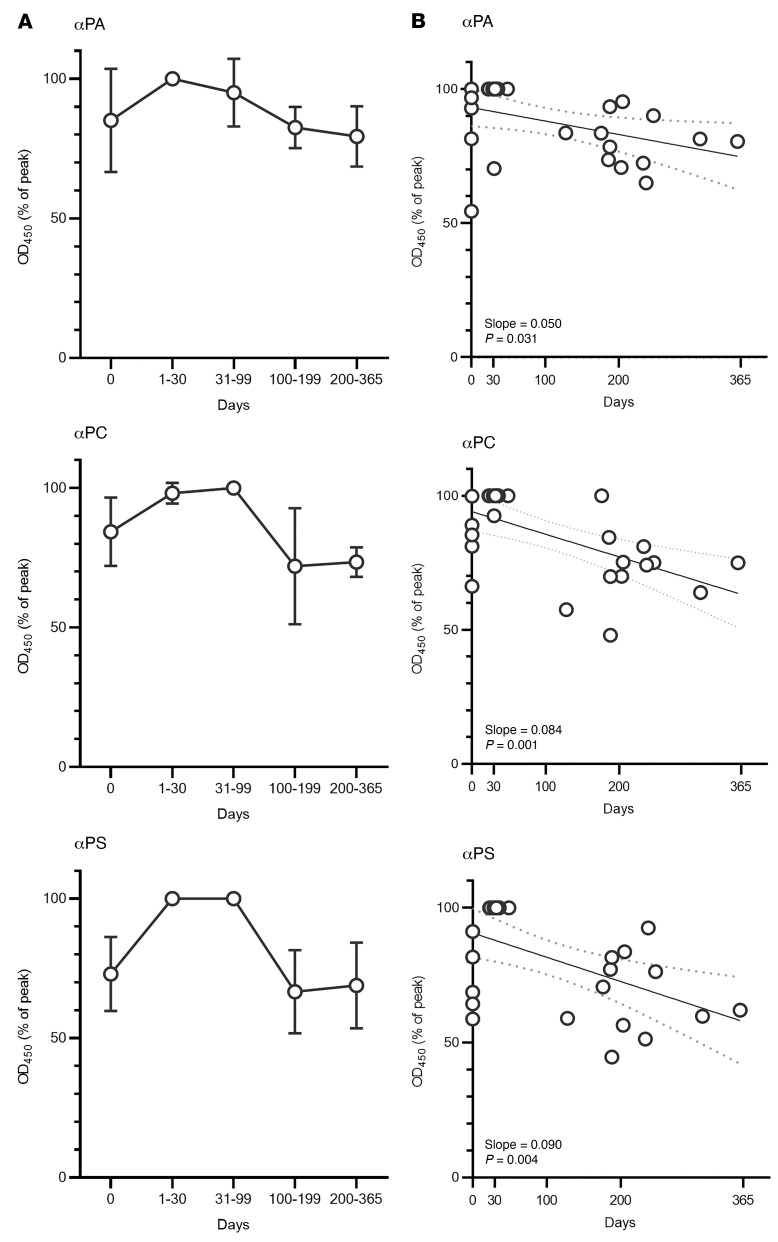
Antibody titers decline after treatment. A group of 10 individuals were sampled serially (2, 3, or 4 times) at time points 0–365 days from the beginning of treatment. Antibody titers (anti-PA, anti-PC, and anti-PS) for each patient are shown as percentages of the highest value for each patient/antibody. In **A**, samples are grouped by time point, with *n* = 5 for day 0, *n* = 4 for days 1–30, *n* = 6 for days 31–99, *n* = 5 for days 100–199, and *n* = 7 for days 200–365. Error bars represent the SD. In **B**, the samples were plotted individually against the day of collection, with trend lines shown in black and 95% CIs in gray dashed lines.

In addition to the treated group described above, the kinetics of antibody responses to infection and treatment were studied using serial samples collected before and at various time points after treatment. Ten patients were evaluated, with 2, 3, or 4 samples collected for each patient. Five patients had pre-therapy (day 0) samples and all patients had convalescent samples, collected a median of 31 days (range, 23–49 days) from the start of therapy and 46.5 days (range, 30–62 days) from the start of illness. Follow-up samples were collected 4–12 months after the start of antibiotic treatment (Supplemental Table 3). To correct for variation in the absolute titers between patients, the data are presented as a percentage of the maximum titer for each antibody in each patient (Figure 9). Titers of all 3 antibodies peaked within 30 days of treatment and began to decline by day 100 after the beginning of treatment. Anti-PA levels declined the least (to 79% of peak titer at 200–365 days) and the slowest, with a slope of 0.05. More pronounced declines were observed for anti-PC (to 74%, slope = 0.08) and anti-PS (to 69%, slope = 0.09). Anti-PA and anti-PC were on average higher on day 0, suggesting that these antibodies may arise faster than anti-PS.

## Discussion

*Borrelia* are fastidious bacteria requiring a complex culture medium with multiple undefined components for in vitro growth (36, 37). As a result, studies of nutrition and metabolism are difficult and rely on modified media depleted of specific nutrients (38, 39). Here, we developed an organic delipidation process for the complex components of the culture medium (serum, BSA, and yeast extract) and demonstrate that *B*. *burgdorferi* was unable to grow in the delipidated media. Using these media, the lipid content of the medium could be defined through the addition of purified lipids in order to identify the lipids that could support *B*. *burgdorferi* growth. Although the necessity of an external lipid source was predicted by a lack of synthetic enzymes in the *B*. *burgdorferi* genome (1), auxotrophy has not, to our knowledge, previously been demonstrated experimentally. The accessibility of the borrelial membrane to fatty acids was previously demonstrated by incorporation of radiolabeled palmitic and oleic acids; the fatty acid composition of cells was shown to mirror that of their growth medium (12).

The utilization of host-derived fatty acids is a common metabolic shortcut in host-associated growth; even organisms possessing pathways for de novo synthesis can bypass them in the presence of an alternative source (40). In addition to facilitating evolutionary genome reduction, the utilization of host-derived fatty acids may serve to suppress the immune responses of NKT cells. CD1d-mediated activation of NKT cells is dependent on both the polar head and fatty acid tail groups of lipids, with small variations in fatty acid composition profoundly affecting the cellular response (41, 42). The ligand-binding site of CD1d has a particular affinity for acyl chains containing characteristic microbial signatures such as anteiso methyl branches and shorter chain lengths; incorporation of host fatty acids into PG and PC can render them up to 100-fold less immunostimulatory than microbe-derived equivalents (43).

Fatty acids appear to be acquired by diffusion into the *B*. *burgdorferi* membrane (44). The ready accumulation of fatty acids and cholesterol (13) suggests the possibility that other exogenous lipid species may also enter borrelial membranes. This was confirmed here by incorporation of fluorescently labeled phospholipid conjugates into *B*. *burgdorferi* cells. The accumulation and utilization of intact PG and PC molecules presents an alternative pathway for membrane metabolism in environments where fatty acids used for phospholipid synthesis are scarce. In BSK medium, the lipid source is rabbit serum, but in vivo *B*. *burgdorferi* is only transiently found in the bloodstream (45, 46), and so this medium is unlikely to model nutrient availability for the in vivo growth of the pathogen. The plasma concentration in humans of free fatty acids is approximately 200 μM (47), a concentration likely to be sufficient for growth of *B*. *burgdorferi*. In other tissues, however, the fatty acid concentration varies widely (48) and can be 10- or 100-fold lower than that of phospholipids (49). Phospholipids may therefore be the most significant lipid source for *B*. *burgdorferi* during growth in the vertebrate host.

In addition to the canonical *B*. *burgdorferi* membrane phospholipids PG and PC, other (non-borrelial) phospholipids are also able to access the membrane and accumulate in cells. PE, PS, and PA from the medium entered the membrane within 1 hour. PA, PE, and PS are all components of mammalian cell membranes and likely to be encountered by *B*. *burgdorferi* through the course of infection. PA is also likely to be present in *Borrelia* as an intermediate in the synthesis of PG and PC (7), although it is not detected in BSK-cultured cells (6, 9), suggesting this intermediate is short-lived and that significant accumulation requires an external source of PA. In serum (and therefore the BSK medium), the principal phospholipid is PC (at ~2000 μM), but PE is also abundant (435 μM). Other phospholipids are present only at low concentrations (<10 μM; ref. 47). In other tissues, however, the relative concentrations of lipid species varies (49), which may affect the repertoire of lipids available to infecting cells at different sites of infection.

The use of host lipids may be a general property of spirochetes. The membranes of in vivo–cultivated *Treponema pallidum* (12) are significantly more diverse than those of in vitro–cultured *Treponema* *denticola* (50), and include phospholipids for which treponemes have no characterized synthetic pathways. In vivo, the fusion of membrane vesicles derived from host cells is thought to be the mechanism by which cholesterol is acquired by *B*. *burgdorferi* (13). Given their origin, such vesicles are likely to contain a great diversity of lipid species, any of which might incorporate into the borrelial membrane with possible consequences for host immune responses. While only 5 phospholipids were assayed here, it is likely that other lipid classes (e.g., sphingolipids and sterols) will also integrate into the membrane and that — to some extent — the lipid composition of *B*. *burgdorferi* will approximate that of the environment.

Antibodies against the borrelial membrane constituent galactosylcholesterol are well characterized in Lyme disease (14, 33). Given this, and the utility of phospholipids in the diagnosis of the related *T. pallidum*, a screen of phospholipid antibodies was conducted in naive and *B*. *burgdorferi*–infected mice. Antibody responses to phospholipids, including those not synthesized by *B*. *burgdorferi* itself, were raised as early as day 7, with the greatest increases seen within 14 days. During infection, IgG antibodies against all assayed phospholipids were raised, with the exception of CL. Antibodies against galactosylcholesterol, which have been characterized in humans but not previously in mice, were also elevated.

Three of the elevated antibodies in mice were tested for potential diagnostic value in human samples. We chose anti-PA and anti-PC, as they reached the highest titers and had the greatest increase (versus the naive controls) of those assayed in mice. Anti-PS was included as it entered *B*. *burgdorferi* cells most readily, after PC. On the basis of the results from the mouse studies, we also included anti-CL as a potential negative control. Anti-PA, anti-PC, and anti-PS were all elevated in infection. Interestingly, the antibody titers against different phospholipids did not correlate, which indicated a degree of independence in the responses. It is unclear whether this reflects localization of the organism to specific tissues, in which *B*. *burgdorferi* may have scavenged different phospholipids, as our sample size was too small to separate patients by manifestation. However, this independence may be valuable for the development of diagnostics if the different antibodies correlate with different clinical characteristics.

As expected, no significant response to CL was observed in the patients with Lyme disease. IgG anti-CL has been previously described in a minority of patients with Lyme disease (51). However, these antibodies were most common in those with neurological symptoms (52), which were described in 3 of the 12 individuals in our untreated group and 2 of the 12 individuals in our treated group. Lyme disease sera are not thought to cross-react with the CL-based nontreponemal tests for syphilis (34, 53). Two positive tests for anti-CL were seen in patients long after treatment, but the significance of this is unclear. A group of 12 patients with syphilis were also tested for anti-PA, anti-PC, and anti-PS antibodies. No significant difference between control and syphilis sera was found for any of the 3 antibodies.

Although all the phospholipids assayed may be present (constitutively or conditionally) in the borrelial membrane, the source of the antigens that precipitate antibody production was undetermined here. The antibodies may be part of a specific adaptive response: other borrelial lipid antigens are presented to T cells by CD1d after phagocytosis (54, 55), and a humoral response to lipid antigens has been demonstrated after infection by other pathogens (26, 56). Alternatively, phospholipid antigens may arise indirectly after host tissue damage during the course of infection (57). In *T*. *pallidum* infection (26), both mechanisms contribute. *B*. *burgdorferi* does not cause tissue destructive lesions comparable to the chancres that develop with syphilis infection, which may account for the lack of an anti-CL response. CL is found predominantly in the mitochondria, and, as such, *B*. *burgdorferi* may not encounter it in significant quantities in the absence of cellular destruction.

Although coinfection of syphilis or relapsing fever spirochetes (such as *Borrelia* *hermsii*) with *B*. *burgdorferi* is unlikely, one limitation of this study is that we did not have access to sufficient sample volumes to test for these or other spirochetal diseases. *Borrelia miyamotoi* coinfections can occur alongside *B*. *burgdorferi* but are currently much less common in the northeastern United States, where our samples were collected. Surveillance of infection rates in ticks in the Northeast typically show 10- to 50-fold less infection with *B*. *miyamotoi* than with *B*. *burgdorferi* (58). *B*. *miyamotoi* infection is treated with the same antibiotics as for Lyme disease, and so possible coinfections would probably also have resolved in our treated group. All patients had Lyme disease, as confirmed by either erythema migrans (EM) rash or STT testing, but we cannot rule out possible coinfections with *B*. *miyamotoi*.

Antibodies against PA and PS were the most reliable diagnostic indicators, identifying 10 of 12 infections and 6 of 12 infections in the untreated group, respectively. Anti-PC was less sensitive, and only 4 individuals in the untreated group passed the positivity cutoff (Table 1). When the antibodies are taken together as a panel, elevation in any 1 of the 3 antibodies diagnosed 11 of the 12 untreated samples. Three of those identified by antiphospholipid antibodies tested negative by conventional STT testing, suggesting that these antibodies may enhance diagnostic coverage early in infection, when the STT test has low sensitivity (59). Further studies with greater numbers of samples will be needed to test this possibility. It is possible that antibodies against phospholipids may arise earlier because of the T cell–independent pathway by which these molecules are processed and activate the immune system. Invariant NKT (iNKT) cells stimulated with lipid antigens interact directly with B cells and may provoke faster activation than peptide-specific T cell interactions do (60, 61).

Two of the 3 antibodies that were elevated prior to treatment declined significantly in the post-treatment samples, and using the same diagnostic cutoff, only 4 of the 12 samples from the post-treatment group were positive for anti-PL antibodies (Table 2). In the same group, all 12 individuals remained positive by STT testing, despite no evidence of new infection. Again, the difference in the processing of phospholipids and the generation of antibodies has been suggested as the reason why antiphospholipid antibodies may be short-lived in the absence of continued stimulation. Although cognate iNKT help of B cells generates a robust initial response, it may not result in the generation of memory B cells (62). In 2 other long-term infections, *Mycobacterium tuberculosis* (19) and *Helicobacter pylori* (63), antiphospholipid antibodies are found to decline after successful treatment. A limitation of these post-treatment samples is that they were collected from patients under evaluation for nonspecific symptoms long after their initial diagnosis and treatment; other conditions unrelated to Lyme disease may have been present. Although none had clinical signs of reinfection, the lack of a gold-standard test for the eradication of *B*. *burgdorferi* makes it impossible to be certain that the treated patients were free of infection at the time of sampling. These factors combined may account for the 4 positive results in the treated group.

To better assess the evolution in antiphospholipid titers during treatment, serial samples were analyzed in a group of 10 patients during treatment and recovery. Although true baseline (pre-infection) titers were not available for these patients, a marked decline was observed in antibody titers over the period of 1 year. This decline was more pronounced for anti-PC and anti-PS titers, suggesting that these antibodies may be of greatest value in monitoring the response to treatment — similar to the use of the nontreponemal tests used to track responses to therapy in syphilis.

Two major gaps in Lyme disease diagnostics are the inability to identify whether continued infection may play a role in post-therapy symptoms and the inability to identify patients with repeat infection. A return to baseline of antiphospholipid antibody levels during convalescence would enable repeat testing if a new or unresolved infection is suspected, in comparison with standard ELISA and Western blot tests, which can remain positive for decades after infection (31). Here, anti-PA and anti-PC antibodies were found to reach approximately 85% of their maximum titer on the day of diagnosis, highlighting their potential application for diagnosis early in the course of infection. This may partly be due to the distinct processing of lipid antigens but may also be a function of their status as autoantibodies. Stimuli driving derepression of existing autoantibodies, already circulating at low levels, may increase titers more rapidly than occurs with antibodies generated de novo by uniquely bacterial antigens.

Previous screens for antibodies raised during *Borrelia* infection have been genomic (64, 65), proteomic (66, 67), or peptide based (68) and therefore neglect the lipid component of cells. The identification of IgG antiphospholipids — in addition to the known anti-galactosylcholesterol (14) — expands the repertoire of antibodies that may have diagnostic potential. Further investigation of the antibodies described here (and potentially other anti-lipids) may reveal them to be valuable diagnostic tools. The prevalence of these antiphospholipid antibodies in the healthy population and in other infectious and inflammatory diseases will need to be determined to understand the specificity of the responses identified here. Given the presence of antiphospholipid antibodies in numerous infectious and inflammatory diseases, it is possible that the responses may lack the specificity required for broad use as a standalone test for Lyme disease. Nevertheless, antiphospholipid antibody testing could be a valuable adjunctive test to standard antibody testing, one that is useful for identifying early infection, repeat infection, and response to therapy.

## Methods

### Culture conditions and delipidated growth medium.

*B. burgdorferi* B31 was routinely cultured in BSK medium composed of BSA (50.00 g/L), CMRL-1066 (9.80, US Biologicals), HEPES (6.60), peptone (5.60), dextrose (5.60), sodium bicarbonate (2.44), yeast extract (2.20), sodium pyruvate (1.00), sodium citrate (0.90), *N*-acetyl glucosamine (0.50), and 6.2% rabbit serum. Media were filter-sterilized and the pH adjusted to 7.6 before the addition of gelatin to 1.4% and sterile water to 1 L, followed by storage at –20°C. The *B*. *burgdorferi* B31 isolate used throughout this study was an infectious strain, cultured from a mouse. By PCR plasmid typing, this strain possessed all B31 plasmids except lp5, cp32-6, and cp32-9, which are not known to carry genes related to either virulence or metabolism.

dBSK was prepared as described above except for the omission of gelatin, which is not essential (36). Defined components were combined as a 5× stock and stored at –20°C. BSA, serum, and yeast extract were mixed to 2× in 50 mL, to which an equal volume of 2:1 chloroform/methanol was added and stirred for 90 minutes. Organic and aqueous phases were separated by centrifugation at 800*g* for 10 minutes. The aqueous phase was retained and underwent 2 further identical extractions. Residual methanol was evaporated overnight under a flow of air, and the 2× delipidated mixture was made to complete medium by addition of the defined stock solution and water to 100 mL, followed by pH adjustment to 7.6 and filter sterilization. The dBSK was stored at –20°C.

For growth curves, fatty acids and cholesterol (Avanti) were stored in chloroform at 50 mM. Lipids at the requisite concentrations were added to 1.5 mL microfuge tubes and the chloroform evaporated under vacuum before resuspension in culture medium. *B*. *burgdorferi* was added to a density of 1 × 10^5^ from a stationary phase culture and incubated at 32°C. Cells were directly enumerated under darkfield microscopy using a Petroff-Hausser counter (Hausser Scientific) daily for 10 days and diluted in PBS at later time points.

### Incorporation of fluorescent lipid analogs.

Cells were grown to a stationary phase (5 days) in BSK and then pelleted (5 minutes at 3220*g*), washed once in an equal volume of PBS, and resuspended in an equal volume of dBSK. The cell suspension (1 mL) was added to nitrobenzoxadiazole-labeled (NBD-labeled) phospholipids (Avanti) to 25 μM. At hourly intervals, the cells were pelleted (5 minutes at 18,800*g*), washed 3 times in 1 mL PBS, and resuspended in 100 μL PBS. The suspensions were transferred onto clear-bottomed, opaque 96-well plates (Corning), and fluorescence was recorded at excitation 460 nm/emission 540 nm (Ex_460_/Em_540_). *Staphylococcus aureus* 502a and *Escherichia coli* MG1655 were grown overnight in tryptic soy broth (TSB): tryptone (17.0 g/L), soytone (3.0), glucose (2.5), sodium chloride (5.0), and dipotassium phosphate (2.5). Incorporation of NBD-phospholipids was carried out in TSB, with subsequent washing and measurement as above.

After fluorescence measurements, 5 μL cell suspensions were spotted onto glass slides and fixed with 5 μL ProLong Gold (Thermo Fisher Scientific) with DAPI. The slides were imaged using a Leica SP8 Falcon with line-sequential imaging of DAPI (Ex = 405 nm, detector = 410–466 nm) and NBD (excitation = 470 nm, detector 550–600 nm) to avoid crosstalk. After acquisition, the images were enhanced using the Leica LAS X software package. Images brightness and contrast were enhanced to illustrate both the fluorescent dye and DAPI staining, with untreated controls processed in the same manner as the experimental images. For all 3 bacterial species imaged, the laser power was calibrated such that minimal autofluorescence was detected in unlabeled cells prior to imaging of the labeled cells.

### Murine infection and endpoint ELISA.

Six female 8-week-old C3H-HeJ mice (The Jackson Laboratory) were infected s.c. with 1 × 10^4^ *B*. *burgdorferi* B31 in 100 μL sterile PBS. Infection was confirmed by culturing of *B*. *burgdorferi* from an ear-punch biopsy taken on day 14. Serum was collected after euthanasia on day 28. Serum was collected after centrifugation for 2 minutes at 18,800*g* and stored at –20°C. Serial samples were collected from 3 male and 3 female 8-week-old mice (The Jackson Laboratory) infected as above, with blood drawn weekly by submandibular punch. Serum was collected from blood as described above.

Hydrophobic Immulon-B–coated, flat-bottomed 96-well plates (Thermo Fisher Scientific) were coated with 1 μg lipid in 100 μL ethanol, which was evaporated overnight. The coated plates were washed twice with 200 μL PBS and blocked for 3 hours at room temperature with 150 μL 1% BSA in PBS, which was then removed by 3 further washes with PBS. Sera were stored diluted in PBS and then serially diluted in 2-fold steps. Sera were incubated at room temperature for 2.5 hours, along with 6 or 12 PBS-only controls per plate. After 3 washes (aspirated by pipetting) with 200 μL PBS plus 0.1% Tween-80, 100 μL goat anti–mouse IgG (H+L, Thermo Fisher Scientific, A16066) at 1:2000 in PBS was added to each well and incubated for 1 hour. Plates were washed 3 times with PBS plus 0.1% Tween-80. One hundred microliters TMB peroxidase substrate (KPL SureBlue, SeraCare) per well was incubated for 5–10 minutes until color developed, and the reaction was stopped with 100 μL 1N HCl. OD_450_ was recorded in a Biotek Synergy HT plate reader; the endpoint titer was the highest dilution at which the average OD_450_ of 3 technical replicates remained higher than the greatest of 12 serum-free controls plus their SD.

### Patient samples.

Discarded human sera from 24 patients undergoing evaluation for Lyme disease were collected, and their medical records were reviewed. The duration of infection was determined from the self-reported date of onset of symptoms (1–75 days prior to sample collection). Untreated patients had the diagnosis of Lyme disease established by the presence of erythema migrans (documented by a clinician) or by facial palsy or arthritis confirmed by a positive STT test. For the untreated patient group, samples were collected prior to antibiotic treatment. Patients in the post-treatment group were all at least 1 year from the onset of infection and initial treatment. Although some individuals had continuing, nonspecific symptoms, none in the post-treatment group had evidence of a new infection: those with a new EM or tick exposure were excluded. Control sera were either collected from healthy volunteers or purchased from SeraCare, which collected the sera from healthy individuals residing in Florida, where Lyme disease is not endemic.

Syphilis sera were purchased from Precision Biomedicine, which sourced the sera from patients positive by both treponemal (syphilis IgG) and nontreponemal (RPR) tests. Available patient data for all groups is included in Supplemental Tables 1–4.

### Single-dilution ELISA.

For single-dilution ELISAs, Immulon-B–coated, round-bottomed 96-well plates (Thermo Fisher Scientific) were coated, washed, and blocked as described above. Sera were diluted to 1:25,000 in PBS and incubated on plates (in triplicate) for 2.5 hours. Five washes with 200 μL PBS plus 0.1% Tween-80 were performed by inversion of the plate, after which 100 μL goat anti–human IgG (H+L, Thermo Fisher Scientific, A18805) at 1:2000 (0.5 μg/mL) in PBS was added. Plates were washed 5 times in 200 μL PBS plus 0.1% Tween-80 and developed with 100 μL TMB peroxidase substrate for 5 minutes exactly, after which the reactions were stopped with 100 μL 1N HCl and read as above. All values were normalized to the average of at least 6 naive controls run on the same plate to yield an anti-PL index, where an index of greater than 1 indicated a titer above the mean of the controls, and less than 1 indicated a titer below this mean. Three technical replicates for each sample were averaged, and the data presented are the combined average of data from 3 independent plates. The cutoff value for positivity was the mean of the naive control group plus 2.291 SDs. This was determined using the statistical method of Frey et al. (35) for a group of 12 samples and a 97.5% CI.

### Statistics.

Simple linear regression analyses (for correlations between single-dilution antibody titers and titer versus collection date), Tukey’s tests (for multiple comparisons), and unpaired, 2-tailed *t* tests (for endpoint and single-dilution ELISAs) were performed using GraphPad Prism, version 9 (GraphPad Software).

### Study approval.

The animal experiments were conducted under the supervision of the IACUC of Tufts University, an institution approved by the Office of Laboratory Animal Welfare at the NIH and accredited by the Association for Assessment and Accreditation of Laboratory Animal Care (International) (AAALAC). Serial serum samples from patients with single or multiple erythema migrans lesions were obtained under a clinical protocol (ClinicalTrials.gov identifier NCT00028080). The study was approved by the IRB of the NIAID, NIH. Written informed consent was obtained from all participants. All patients fulfilled the US Centers for Disease Control and Prevention 2017 Lyme Disease case definition criteria.

## Author contributions

LTH and PJG designed experiments, analyzed data, and wrote the manuscript jointly. Experimental data were collected by PJG and LHC. ARM and SPT contributed to clinical data acquisition, data interpretation, and drafting of the manuscript. All authors assisted in revising the manuscript.

## Supplementary Material

Supplemental data

## Figures and Tables

**Table 1:**
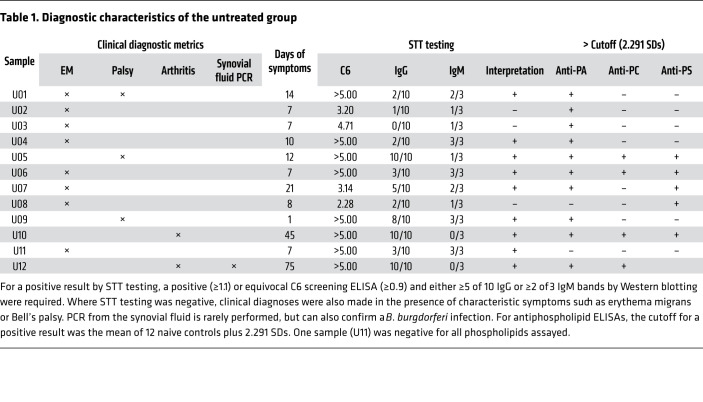
Diagnostic characteristics of the untreated group

**Table 2:**
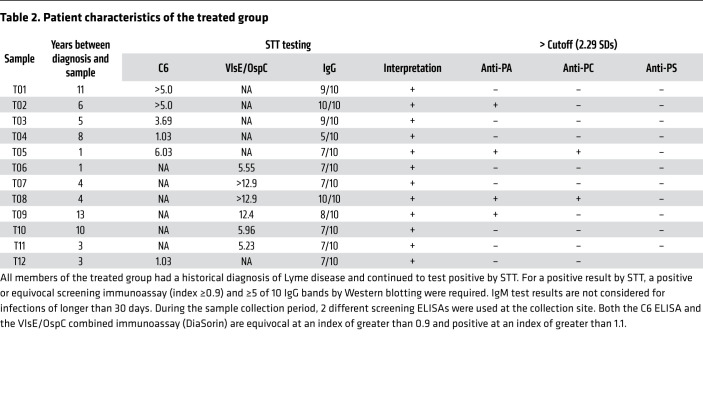
Patient characteristics of the treated group

## Version 1 03/15/2022

Electronic publication
