# Supplementary material for: Antiphospholipid autoantibodies in Lyme disease arise after scavenging of host phospholipids by Borrelia burgdorferi
Source: J Clin Invest. 2022 Mar 15;132(6):e152506. doi: 10.1172/JCI152506 (PMC8920326; doi:10.1172/JCI152506)
Supplement: Supplemental data [file jci-132-152506-s052.pdf]

Sample	Age	Sex	Symptoms						Clinical Diagnostic Metrics				Days of symptoms	STT Testing			> Cutoff (2.291 SD)			
			Arthralgia	Fatigue	Nausea	Fever	Myalgia	Headache	EM	Palsy	Arthritis	PCR		C6	IgG	IgM	αPA	αPC	αPS	αCL
U01	48	F	•	•					•	•	-	N/A	14	>5.00	2/10	2/3	+	-	-	-
U02	58	M			•				•	-	-	N/A	7	3.20	1/10	1/3	+	-	-	-
U03	35	F				•			•	-	-	N/A	7	4.71	0/10	1/3	+	-	-	-
U04	34	F		•					•	-	-	N/A	10	>5.00	2/10	3/3	+	-	-	-
U05	50	M			•			•	-	•	-	N/A	12	>5.00	10/10	1/3	+	+	+	-
U06	61	M		•			•	•	•	-	-	N/A	7	>5.0	3/10	3/3	+	+	+	-
U07	71	M				•			•	-	-	N/A	21	3.14	5/10	2/3	+	-	+	-
U08	19	M							•	-	-	N/A	8	2.28	2/10	1/3	-	-	+	-
U09	64	M							-	•	-	N/A	1	>5.00	8/10	3/3	+	-	-	-
U10	12	F	•						-	-	mono-articular	N/A	45	>5.00	10/10	0/3	+	+	+	-
U11	42	M	•		•			•	•	-	-	N/A	7	>5.00	3/10	3/3	-	-	-	-
U12	43	M	•						-	-	mono-articular	•	75	>5.00	10/10	0/3	+	+	+	-

Table SI1. Characteristics of the Untreated group

For a positive result by standardized two-tier (STT) testing, a positive (≥1.1) or equivocal C6 screening ELISA (≥0.9) and either ≥5/10 IgG or ≥2/3 IgM bands by Western blot are required. Where STT testing is negative, clinical diagnoses are also made in the presence of characteristic symptoms such as the erythema migrans or Bell's palsy. PCR from the synovial fluid is rarely performed, but can also confirm a *B. burgdorferi* infection. For antiphospholipid ELISAs, the cutoff for a positive result was the mean of 12 naïve controls + 2.291 standard deviations. One sample (U11) was negative for all phospholipids assayed. Mean age 44.8 years, median 45.5.

Sample	Age	Sex	Symptoms at sample date								Years (diagnosis to sample)	STT Testing			> Cutoff (2.291 SD)		
			Arthritis	Arthralgia	Memory	Neuropathy	Headache	Fever	Myalgia	Fatigue		C6	VlsE/OspC	IgG	αPA	αPC	αPS
T01	52	F	mono-articular	•							11	>5.0	N/A	9/10	-	-	-
T02	47	M	pauci-articular								6	>5.0	N/A	10/10	+	-	-
T03	68	M		•	•						5	3.69	N/A	9/10	-	-	-
T04	62	F			•						8	1.03	N/A	5/10	-	-	-
T05	52	F				•					1	6.03	N/A	7/10	+	+	-
T06	34	M		•		•					1	5.55	N/A	7/10	-	-	-
T07	31	M								•	4	N/A	>12.9	7/10	-	-	-
T08	36	M	mono-articular					•		•	4	N/A	>12.9	10/10	+	+	-
T09	69	M		•	•		•				13	N/A	12.4	8/10	+	-	-
T10	37	M		•	•						10	N/A	5.96	7/10	-	-	-
T11	23	M		•	•				•	•	3	N/A	5.23	7/10	-	-	-
T12	77	M								•	3	1.03	N/A	7/10	-	-	-

Table SI2. Characteristics of the Treated group

STT testing is performed as above, but IgM data are discounted where symptoms have persisted for >30 days. During the sample collection period, two different screening ELISAs were used at the collection site. Both the C6 ELISA and the VlsE/OspC combined immunoassay (DiaSorin) are equivocal at an index >0.9 and positive at >1.1. Mean age 49.0 years, median 49.5.

Patient	Age	Sex	EM	Sample	Days since		STT Testing			% of peak value		
					Symptoms	Treatment	C6	IgG	IgM	αPA	αPC	αPS
A	50-59	F	•	A000	1	0	0.679	N/A	N/A	92.9	66.3	58.8
				A049	50	49	5.71	2/10	2/3	100.0	100.0	100.0
				A205	204	205	3.58	2/10	1/3	95.3	75.2	83.7
B	50-59	F	•	B000	11	0	4.12	3/3	3/3	54.5	81.2	81.7
				B036	47	36	8.76	3/10	3/3	100.0	100.0	100.0
				B203	214	203	1.56	2/10	2/3	70.7	70.0	56.5
C	20-29	F	•	C000	15	0	8.22	0/10	2/3	100.0	89.1	91.3
				C031	46	31	10.35	2/10	2/3	70.4	100.0	100.0
				C186	201	186	3.62	0/10	2/3	73.7	84.5	77.2
D	40-49	F	•	D023	30	23	8.03	2/10	3/3	100.0	100.0	100.0
				D128	135	128	6.93	2/10	2/3	83.7	57.6	59.0
				D310	317	310	3.47	2/10	1/3	81.4	64.0	59.8
E	40-49	F	•	E024	62	24	9.01	5/10	3/3	100.0	100.0	100.0
				E247	285	247	NDA	NDA	NDA	97.6	80.9	65.6
F	40-49 F	F	•	F000	1	0	0.19	N/A	N/A	96.8	99.9	68.7
				F030	31	30	1.03	4/10	3/3	100.0	92.6	100.0
				F176	177	176	0.35	N/A	N/A	83.6	100.0	70.6
G	40-49	F	•	G033	55	33	11.03	3/10	3/3	100.0	100.0	100.0
				G158	180	158	7.35	0/10	3/3	78.4	48.0	44.7
H	50-59	F	•	H035	50	35	10.03	3/10	3/3	100.0	100.0	100.0
				H233	248	233	3.35	3/10	2/3	72.4	81.1	51.4
I	50-59	M	•	I029	38	29	11.05	-	+	100.0	100.0	100.0
				I246	246	237	3.71	4/10	3/3	65.0	74.1	92.6
J	50-59	M	•	J000	6	0	0.55	N/A	N/A	81.5	85.5	64.4
				J032	38	32	0.63	N/A	N/A	100.0	100.0	100.0
				J188	194	188	0.62	N/A	N/A	93.5	69.9	81.6
				J361	367	361	0.55	N/A	N/A	80.5	75.0	62.0

Table SI3. Patient data for serial samples

Data from 10 patients diagnosed with Lyme disease by the presence of an erythema migrans rash (EM). Multiple (2-4) samples were taken from each patient at different time points up to 1 year after the beginning of treatment. D0 (pre-treatment) samples were available for 5 patients. Antiphospholipid titers are shown as a percentage of each individual's peak value as measured by optical density at a single dilution. NDA = no data available. N/A indicates testing was not performed; where the C6 ELISA is negative second tier testing is not carried out. For one sample, only the interpretation (+/-) and not the full Western blot data were available.

Sample	Age	Sex	Syphilis IgG		RPR test	aPL results		
			Result	Interpretation	Result	αPA	αPC	αPS
S01	58	M	2.1	positive	reactive	+		
S02	50	M	> 8.0	positive	reactive			
S03	44	M	> 8.0	positive	reactive			
S04	40	F	> 8.0	positive	reactive			
S05	40	M	> 8.0	positive	reactive			
S06	49	F	> 8.0	positive	reactive			
S07	46	F	> 8.0	positive	reactive			
S08	28	F	> 8.0	positive	reactive			
S09	43	M	> 8.0	positive	reactive			
S10	45	F	> 8.0	positive	reactive			
S11	49	M	> 8.0	positive	reactive			
S12	50	M	1.8	positive	reactive	+		

Table SI4. Characteristics of the Syphilis group

Syphilis is diagnosed by a treponemal test for protein antigens and a nontreponemal test for lipid antigens. The treponemal IgG test (Bioplex 2200) is positive at an index >1. The nontreponemal test used at this collection site (ASI card test) gives only a qualitative reactive/unreactive result. For antiphospholipid ELISAs performed here, the cutoff for a positive result was the mean of 12 naïve controls + 2.291 standard deviations. Syphilis sera were purchased from Precision Biospecimens.

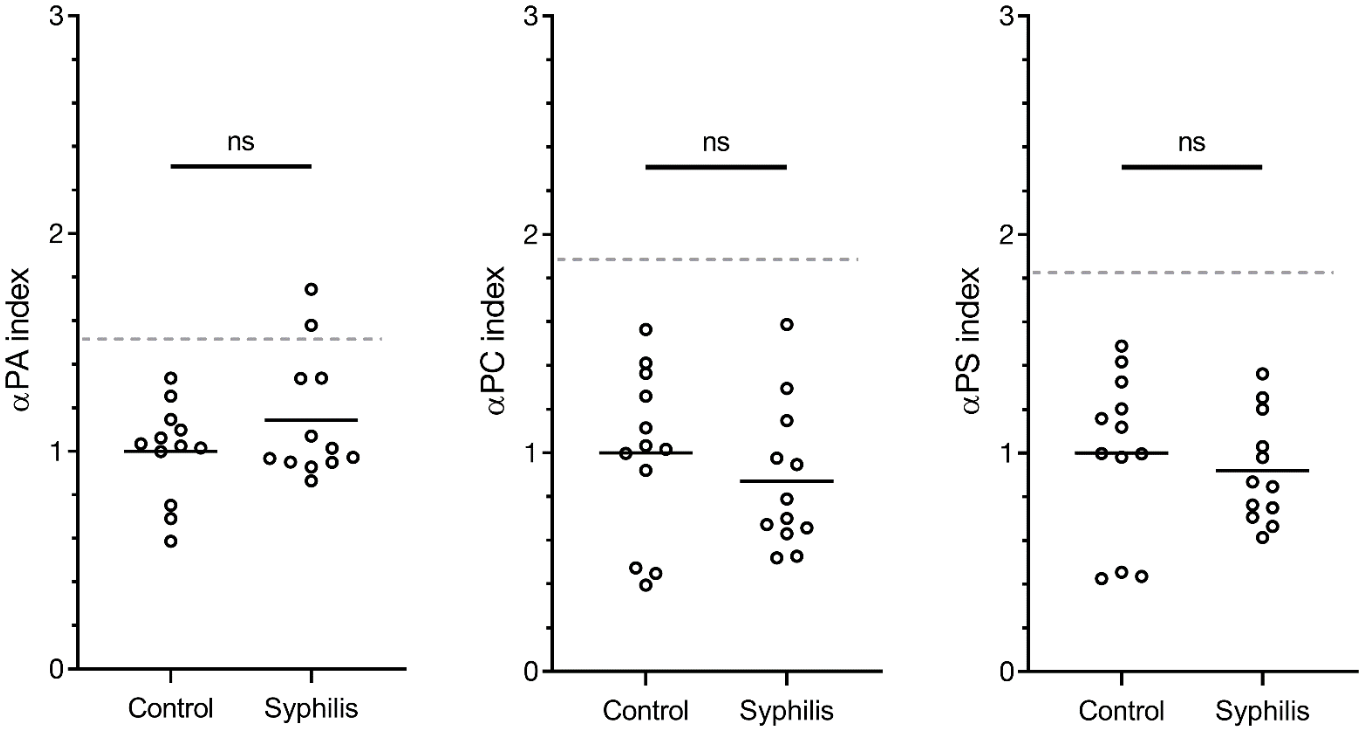

Figure S1. Antiphospholipid indices in Syphilis (*Treponema pallidum*) infection.

12 syphilis sera were tested for antiphosphatidic acid (αPA), antiphosphatidylcholine (αPC), and antiphosphatidylserine (αPS). None of the antibody titers were significantly different in syphilis and healthy control sera. Black lines represent the mean of each group. An index of >1 indicates antibody titer was above the level of the naïve controls. Grey dashed lines represent the cutoff value (mean N + 2.291 standard deviations) above which a sample was considered positive. Significance calculated by unpaired 2-tailed t test, where $p > 0.05$ is nonsignificant (ns).
